# Trends in Kaposi's Sarcoma in Miami Beach from 1987 to 2007

**DOI:** 10.5402/2012/642106

**Published:** 2012-12-25

**Authors:** Simon B. Zeichner, Ana L. Ruiz, Gabriel P. Suciu, Rachel Lerner Zeichner, Estelamari Rodriguez

**Affiliations:** ^1^Department of Internal Medicine, Mount Sinai Medical Center, 4300 Alton Road, Miami Beach, FL 33140, USA; ^2^Department of Hematology and Oncology, Mount Sinai Medical Center, Miami Beach, FL 33140, USA; ^3^Department of Public Health and Biostatistics, College of Osteopathic Medicine, Nova Southeastern University, Fort Lauderdale-Davie, FL 33314, USA; ^4^Department of Clinical Psychology, Nova Southeastern University, Fort Lauderdale-Davie, FL 33314, USA

## Abstract

*Purpose.* Kaposi's sarcoma (KS) is a rare low-grade vascular tumor associated with the human herpes virus 8. By analyzing the epidemiology, staging, and treatment of KS, we hoped to improve the quality of care at our institution. *Methods*. Review of the Mount Sinai Medical Center tumor registry database in Miami Beach, FL, USA, identified 143 cases of KS between January 1, 1987 and December 31, 2007. *Results.* The majority of patients were non-Hispanic whites, non smoking males diagnosed between 1987 and 1996. Most of the patients were HIV positive, with an equal percentage diagnosed with local or distant disease. Most patients received no chemotherapy or radiation. There were no significant differences in patient survival based on sex, HIV status, or radiation received. There was a trend toward improved survival among older patients who smoked, received no chemotherapy, and had localized stage at diagnosis. Multivariate analysis revealed that non-Hispanic whites had a significant worse survival than Hispanic whites (HR = 0.55, 95% CI (0.33, 0.90), *P* = 0.02). Patients diagnosed between 1987 and 1996 had a worse survival than those between 1997 and 2007 (HR = 0.33 (95% CI 0.19, 0.55), *P* < 0.0001). *Conclusion.* This large retrospective study provides further insight into KS. Ethnicity and date of diagnosis are important predictors of long-term survival.

## 1. Introduction

First described by Hungarian dermatologist Kaposi in 1872 [1], Kaposi's sarcoma (KS) is a rare low-grade, vascular tumor associated with the human herpes virus 8 (HHV-8) [2]. KS is divided into four epidemiological subtypes: (a) classic (indolent cutaneous proliferative disease affecting older men of Mediterranean and Jewish origin [3, 4]), (b) endemic (from Africa [5, 6]), (c) solid organ transplant associated, and (d) epidemic (acquired immunodeficiency syndrome (AIDS) associated [7]). Kaposi's sarcoma is 15 times more prevalent among men than women and is most common among homosexual males. Risk factors for tumor development include human immunodeficiency virus (HIV) infection [8–10], high anti-HHV-8 antibody titers [10], HHV-8 viremia [11], inherited variation in immuno-modulating genes [12], immunosuppression with steroids or other immuno-suppressants [13], and absence of tobacco use [13–15].

Kaposi's sarcoma typically presents as cutaneous purplish, reddish-blue, or dark brown/black macules, plaques, and nodules on the lower legs and feet with or without associated extremity lymphedema. Kaposi's sarcoma lesions are heterogeneous with respect to both size and aggressiveness. Although most cases follow a chronic indolent course [16–18], KS occasionally presents with a more aggressive, rapid course. Extracutaneous involvement of the mucous membranes, gastrointestinal tract, regional lymph nodes, and respiratory tract can occur during the course of the disease, particularly in the AIDS-associated subtype. Involvement of the oral cavity occurs in approximately one-third of patients and is the site of initial presentation in roughly 15 percent. In the era prior to highly active antiretroviral treatment (pre-HAART), a higher rate of involvement of the GI tract was reported, with 40 percent at initial diagnosis and 80 percent at autopsy.

The demographics, epidemiology, diagnosis, and treatment for Kaposi's sarcoma have changed significantly over the past 30 years with the spread of the HIV/AIDS epidemic in the early 1980s, the widespread introduction of combination highly active antiretroviral treatment (HAART) in the mid-1990s, and the advancing age of the United States population in the 2000s. The Miami Beach community had a unique position during this time span; it served a large group of elderly patients while also serving a population that was one of the epicenters for the HIV/AIDS epidemic in the United States [19, 20].

In order to further understand this rare disease and its corresponding staging, treatment, and prognosis, we analyzed a large case series at our institution. With the knowledge gained from this study, we hoped to improve the quality of care at our institution.

## 2. Materials and Methods

### 2.1. Patient Population

This retrospective study was conducted with prior approval of the Mount Sinai Medical Center Institutional Review Board. We reviewed the tumor registry of Mount Sinai Medical Center in Miami Beach, FL, USA to identify all cases of Kaposi's sarcoma diagnosed between January 1, 1987 and December 31, 2007. There were 143 cases identified according to the International Classification of Diseases-Oncology, 1st, 2nd, and 3rd Editions (ICD-O-1: 1976–1989, ICD-O-2: 1990–2000, ICD-O-3: 2001–2007 [21]). Patients' data included sex, ethnicity, age at diagnosis, date of diagnosis, smoking status, site of KS presentation, stage of disease at diagnosis (Surveillance Epidemiology and End Results (SEER) local/regional/distant classification scheme [22]), type of treatment received (chemotherapy, radiation), and HIV status (Table 1). No patients were excluded from this analysis.

### 2.2. Statistical Analysis

Descriptive statistics were used for analysis of clinical variables. The chi-square test of independence was used to assess associations between categories. A time-to-event analysis was constructed to evaluate overall survival (OS) in relation to clinical category. Survival time was defined as the period from date of diagnosis until date of death or date of last visit. Patients were classified into two categories based on their survival status: censored or death. The Kaplan-Meier [23] and Cox proportional hazards methods [24] were used to estimate the overall survival and create a prognostic factor model based on the significant covariates: age (>50 versus ≤50), year of diagnosis (1997–2007: post-HAART versus 1987–1996: pre-HAART), sex (male versus female), self-designated ethnicity/race (Hispanic whites, non-Hispanic whites, black), smoking status (current versus former versus nonsmoker), site of presentation (skin versus nonskin), stage (local versus regional versus distant), chemotherapy (chemotherapy versus no chemotherapy), radiation (radiation versus no radiation), and HIV status (HIV-positive versus HIV-negative). The hazard ratios (HR) were estimated for risk of death due to various covariates in the model [25]. For the survival curve comparisons, the log-rank or Wilcoxon-Breslow tests were used when the proportional hazards assumption was not violated. Otherwise, the Fleming-Harrington or Peto & Peto-Prentice tests were used [26]. All tests were two-sided, and the type I error was considered to be 0.05. The SPSS, SAS, and STATA statistical packages were used for all analyses.

## 3. Results

Of the 143 KS patients identified, the majority were males (90.2%), non-Hispanic whites (60.1%) nonsmokers (42.7%) who were diagnosed between 1987 and 1996 (57.3%, Table 1). The median age at diagnosis was 41.0 years and ranged from 26 to 96. The greatest percentages of patients were diagnosed between ages 21–40 (45.5%), followed by ages 41–60 (29.4%), and >80 (14.0%). The most common site of initial presentation was the skin (87.4%) and an equal percentage of patients had local (40.6%) and distant disease (40.6%) at diagnosis. Most of the patients were HIV-positive (52.4%) and received neither chemotherapy (80.4%) or radiation therapy (65%).

**Figure 1:**
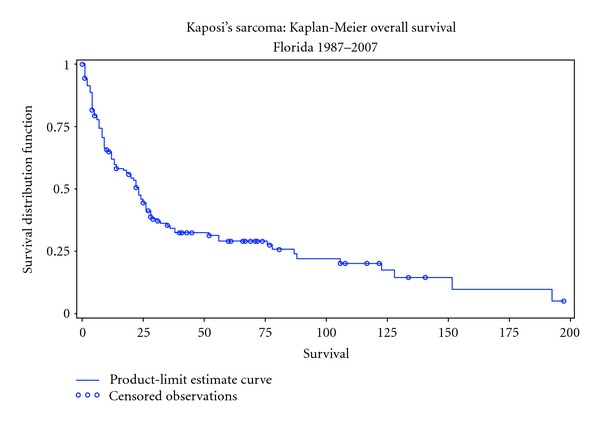
Kaplan-Meier curve demonstrating the overall survival (27% at 5 years) in a population of Kaposi's sarcoma patients diagnosed at Mount Sinai Medical Center in Miami Beach, FL, USA.

**Figure 2:**
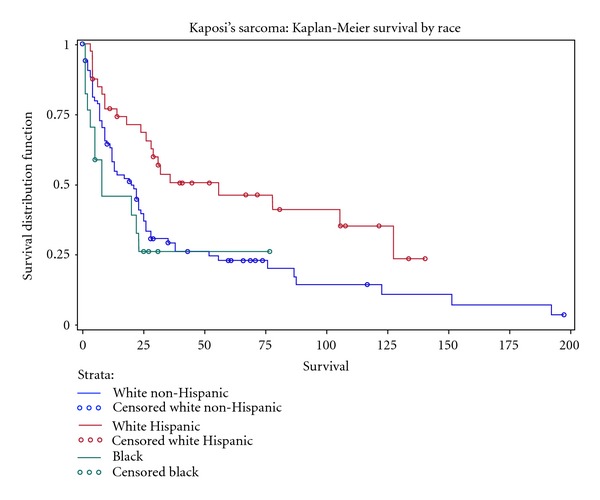
Kaplan-Meier curve demonstrating a significantly improved survival based on ethnicity (non-Hispanic white versus Hispanic white, *P* = 0.01) in a population of Kaposi's sarcoma patients diagnosed at Mount Sinai Medical Center in Miami Beach, FL, USA.

**Figure 3:**
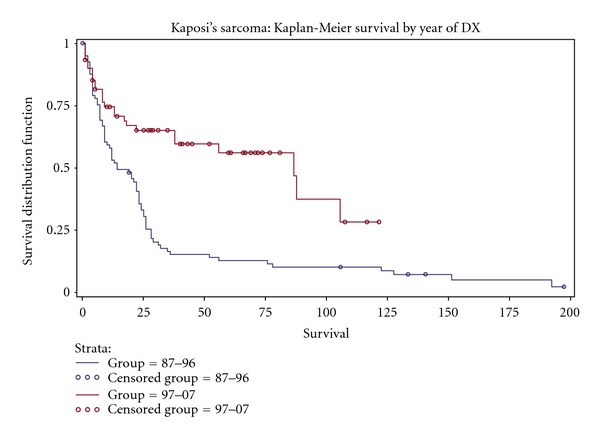
Kaplan-Meier curve demonstrating a significantly improved survival based on date of diagnosis (1997–2007 versus 1987–1996, *P* = 0.004) in a population of Kaposi's sarcoma patients diagnosed at Mount Sinai Medical Center in Miami Beach, Florida.

**Figure 4:**
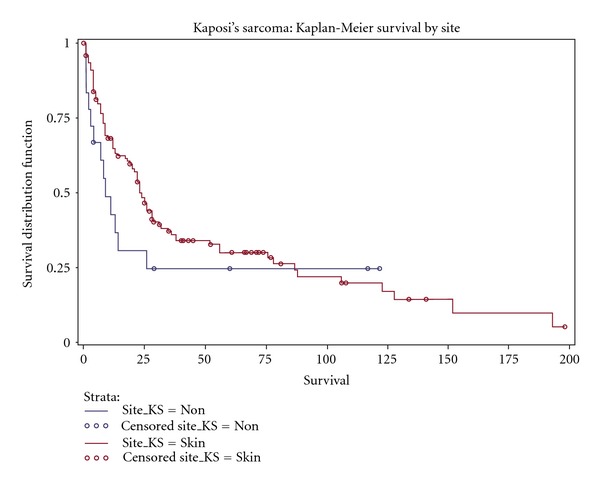
Kaplan Meier curve demonstrating a significantly improved survival based on site of initial presentation (skin versus non-skin; *P* = 0.04) in a population of Kaposi's sarcomas patients diagnosed at Mount Sinai Medical Center in Miami Beach, Florida.

The Kaplan-Meier analysis demonstrated an overall survival of 27% at 5 years (Figure 1) with a median survival of 24 months. There was no significant difference in survival among patients based on sex (28 versus 23 months, females versus males, resp.; *P* = 0.84), HIV status (19 versus 25 months, HIV-positive versus HIV-negative, resp.; *P* = 0.91), or radiation therapy (23 versus 22 months, no radiation versus radiation, resp.; *P* = 0.98) (Table 2). There was a significant difference in survival based on ethnicity/race (20 versus 56 versus 8, non-Hispanic whites versus Hispanic whites versus blacks, resp.; *P* = 0.01) (Figure 2), date of KS diagnosis (14 versus 87 months, 1987–1996 versus 1997–2007, resp.; *P* = 0.004) (Figure 3), and site of initial presentation (23 versus 9 months, skin versus nonskin, resp.; *P* = 0.04) (Figure 4). There was a trend toward a significant difference based on smoking status (23 versus 17 versus 36 versus 15 months, nonsmoking versus ex-smoking versus current smoking versus unknown, resp.; *P* = 0.32), age at diagnosis (22 versus 18 versus 38 versus 32 months, 21–40 versus 41–60 versus 61–80 versus >80, resp.; *P* = 0.34), stage at diagnosis (32 versus 22 versus 17 months, local versus regional versus distant, resp.; *P* = 0.17), and chemotherapy (23 versus 14 versus 9 months, no chemotherapy versus single-agent chemotherapy versus multiple-agent chemotherapy, resp.; *P* = 0.26).

Multivariate analysis revealed Hispanic whites were 45% less likely to die than non-Hispanic whites when controlling for year of diagnosis (Table 3). Patients diagnosed between 1997 and 2007 were 67% less likely to die than the patients diagnosed between 1987 and 1996 when controlling for ethnicity.

## 4. Discussion

### 4.1. Demographic and Epidemiological Data

As with earlier case series and reported epidemiological data [27–40], this study found that while men were nine times more likely than women to develop KS, the five-year survival for men and women was essentially the same. Compared to those KS patients who never smoked, there was a trend toward improved survival among current or former smokers, irrespective of age at diagnosis. This finding supports a putative protective effect for cigarette smoking in KS patients, as has previously been reported [13–15]. Future preclinical studies are needed to further elucidate a mechanism for this unusual finding. Based on this study, newly diagnosed KS patients should have clear documentation in the medical records regarding their smoking status, as this may have prognostic value.

An unexpected finding in our series was the higher five-year survival rate among the Hispanic white population. We initially hypothesized this difference was due to an older non-Hispanic white population with multiple comorbidities and a Hispanic white population with greater access to healthcare, supportive services, and community resources. Upon further data analysis, as opposed to non-Hispanic white patients, a significant majority of the Hispanic white patients were less than 50 years old. Despite a relatively small patient population, we suspect the difference in five-year survival was, in part, due to a younger, healthier Hispanic white KS population. However, an underlying genetic polymorphism contributing to improved survival must be considered, as there is evidence that differences in KS survival among ethnic groups can be explained by a TP53 polymorphism at codon 72 [41]. Future studies should continue to investigate a genetic predisposition for improved survival among Hispanic white patients. In terms of patient care, it seems reasonable to incorporate more intensive treatment regimens for distant disease, particularly in the non-Hispanic white population. From both quality of care and genetic research perspectives, it will be important for physicians and researchers to document specific patient ethnic backgrounds in the medical records.

Consistent with previous literature [3, 4, 7], we observed a bimodal distribution pattern based on HIV status and age at KS diagnosis. The majority of patients fit into one of two categories, young HIV-positive men and elderly HIV-negative men. We attempted to correct for this by dividing patients into two age groups, younger than or equal to age 50 and older than age 50. There was a trend toward improved survival among HIV-negative patients greater than 50 years old, suggesting that these patients had the classic-endemic subtype of KS. These patients would likely die from disease unrelated to KS and could be managed with less aggressive therapy. However, those patients diagnosed before age 50 were generally HIV-positive, had widespread disease at presentation, received multiple treatment regimens, and would likely to die from KS or other AIDS-related diseases. Although larger studies are needed for validation, this study supports the need to treat younger, endemic KS patients more aggressively than older, classic KS patients.

### 4.2. Date of Diagnosis and SEER Data

Similar to earlier studies, the majority of patients in our study were diagnosed between 1987 and 1996, corresponding with the peak of the HIV epidemic [42]. In our study population, there was a significant difference in five-year survival based on date of diagnosis, supporting the widely recognized observation of multiagent HAART therapy revolutionizing the treatment of HIV/AIDS and AIDS-related illnesses [33–40]. Based on the results of our study, it is imperative that patients with HIV-related KS be treated with HAART. Future studies of KS will need to incorporate specific HAART regimens and degree of immunosuppression, as measured by cluster of differentiation 4 (CD4) counts and HIV viral loads, in order to study the effects of particular treatment regimens and determine the relationship between immunosuppression and survival.

### 4.3. Staging

The patients in our study population with distant disease at diagnosis had a significantly shorter survival compared to patients with local and regional disease at diagnosis. However, patients with regional disease had essentially the same survival as those with local disease only. These findings reflect the strengths and limitations of the SEER local/regional/distant KS classification scheme. All of our patients were classified based on this system, which was amended twice during the course of our study period [43]. Using the SEER staging adjustments table, we could accurately interpret all diagnoses. Meanwhile, KS staging has proven difficult over the past 30 years due to the lack of a widely accepted standard staging system. Several classification systems have been proposed including one describing the cutaneous presentation of the disease: Stage I (macular nodular stage), stage II (infiltrative stage), stage III (florid stage), and stage IV (disseminated stage [44]). Another proposed staging system for AIDS-related KS subdivided patients into low- and high-risk groups based on the extent of the tumor, immune status, and severity of systemic illness [45]. Future studies should attempt to incorporate all proposed classifications systems and produce a standard staging system that accurately characterizes disease severity.

### 4.4. Treatment

We hypothesized that those patients given chemotherapy and/or radiation would have longer survival times. However, our data revealed a trend toward worsening survival among patients receiving chemotherapy. This unusual finding may in part be explained by the fact that patients receiving chemotherapy were more likely to have advanced and bulky disease at diagnosis. Since there were no National Comprehensive Cancer Network (NCCN) guidelines for disease management, decisions to treat patients with chemotherapy were left to physician's discretion. Similar to several other malignancies, the major goals of KS treatment involve symptom palliation, prevention of disease progression, and attempts to improve survival [46]. This approach has been implemented due to the inability to fully eradicate latent HHV-8 infection (the main risk factor for the tumor [26–31]). Unlike chemotherapy, radiation is a local therapy often administered for control of symptoms for both local and advanced diseases. Therefore, although all patients were staged for extent of disease, patients with localized, nonbulky disease were more likely to be treated with radiation alone.

Another possible explanation for the lack of significant survival benefit with chemoradiation techniques was the difference in chemotherapy treatment and radiation techniques offered to the patient population. Treatment for KS changed over the past 30 years and patients diagnosed and treated in the 2000s were treated much differently than those in the 1980s and 1990s. Currently, treatment options for localized cutaneous KS lesions include surgery [47], radiation [48, 49], cryotherapy/laser therapy [50–52], intralesional injection of chemotherapy (vinblastine; [53, 54]), topical therapy (*cis*-retinoic acid [55]), and (imiquimod [56]). Highly active antiretroviral treatment (HAART) is recommended for all patients with AIDS-related KS [57–59], despite the finding that its initiation is associated with temporary progression of KS lesions within the first three to six weeks, corresponding with the well-described immune reconstitution syndrome [60, 61]. Despite this observation, HAART is recommended for all patients with KS, as the benefits are thought to strongly outweigh the risks.

Currently accepted indications for systemic chemotherapy for KS include widespread skin involvement, extensive skin involvement unresponsive to other therapies, extensive extremity edema, symptomatic visceral organ involvement, and immune reconstitution syndrome. Pegylated liposomal doxorubicin and liposomal doxorubicin have been established as first-line treatments for KS, unless there is a cardiac contraindication [62]. Highly active antiretroviral treatment (HAART) plus chemotherapy has a significant advantage in treating AIDS-associated KS. One study found that only 20 percent of patients responded to HAART alone, whereas HAART plus pegylated liposomal doxorubicin produced response rates ranging from 50 to 70 percent [63]. Although paclitaxel has been thought to be potentially more toxic than liposomal doxorubicin, it has been established as a second-line treatment for KS [64–71]. Recombinant interferon alpha is another agent approved for the treatment of AIDS-related KS and has produced beneficial responses in 20–40 percent of patients [72, 73].

Based on the results of our study, we believe that each patient's treatment should be individualized, taking into account immune status, tumor bulk, ethnic status, comorbidities, and quality-of-life measures. Large randomized studies are needed to compare currently accepted treatment options. In addition to survival studies incorporating new treatment guidelines [74], the development of specific NCCN guidelines regarding disease management may be of value in limiting treatment variability.

### 4.5. Limitations

One of the major limitations of our study was the inability to obtain CD4 counts and HIV viral loads for HIV-positive patients. We were also unable to obtain patients' HAART treatment history. Due to these limitations, we were unable to reach conclusions regarding KS survival and degree of immunosuppression from HIV/AIDS. From the data obtained from this study, we believe that tumor databases, when dealing with HIV-associated malignancies, need to include information regarding patient's HIV status and treatment. This information will be useful not only for future KS studies, but also for the treatment planning of patients.

Another limitation of our study was our inability to determine cause of death of our patients. Due to this limitation, we were unable to draw conclusions regarding whether patients died from KS versus other disease processes. This information would have been useful in the elderly and post-HAART HIV patients where we suspect many succumbed to disease processes unrelated to KS. 

Our decision to create the timeframes (1987–1996 and 1997–2007) was not arbitrary. The United States Federal Drug Administration approved the first protease inhibitor, saquinavir, on December 6, 1995 [75]. Although approved in late 1995, the drug, and other new protease inhibitors, was not widely accepted or used until mid-1996 [76]. It was at this time that patients began receiving the current standard of care, what we commonly refer to as “triple therapy HAART.” Therefore, although not exact, we believed that the use of 1997 as a starting point for post-HAART therapy was appropriate.

We were unable to report on the type of chemotherapy or radiation used in the treatment of our KS patients. These data were not recorded in the cancer database during the 1980s and 1990s. As was previously mentioned, KS treatment greatly improved over the course of the study timeframe. Therefore, consolidating all radiation and chemotherapy regimens prohibited conclusions regarding efficacy. 

Our study did not distinguish between subtypes of KS, including those associated with organ transplants. However, we assumed that the total number of patients from this group was negligible, because our hospital did not perform solid organ or stem cell transplants.

## 5. Conclusion

This large retrospective study provides further insight into the epidemiology, staging, treatment, and prognosis of KS. The majority of KS patients identified were young, nonsmoking, HIV-positive, non-Hispanic white males diagnosed during the peak of the HIV epidemic. There were no significant differences in survival among patients based on sex, HIV status, or radiation received. There was a trend towards an improved survival among patients who were older, currently smoking, had localized stage at diagnosis, and had received no chemotherapy. Hispanic white patients had superior outcomes compared with non-Hispanic whites. Patients diagnosed from 1997 to 2007 had superior outcomes compared to those diagnosed from 1987 to 1996.

## Figures and Tables

**Table 1:** Epidemiological, staging, and treatment descriptive frequencies in a population of Kaposi's sarcoma patients diagnosed at Mount Sinai Medical Center in Miami Beach, FL, USA.

Category	Median	Standard deviation
Age at diagnosis (years)	41	19.9

Category	Number (*n*)	Percentage (%)

Sex		
Female	14	9.8
Male	129	90.2
Race and ethnicity		
White, non-Hispanic	86	60.1
White Hispanic	40	28.0
Black	17	11.9
Smoking status		
Nonsmoker	61	42.7
Ex-smoker	37	25.9
Current smoker	21	14.7
Unknown	24	16.8
Age range at diagnosis		
21–40	65	45.5
41–60	42	29.4
61–80	16	11.2
>80	20	14.0
Year of Kaposi's diagnosis		
1987–1996	82	57.3
1997–2007	61	42.7
Site of initial presentation		
Nonskin	18	12.6
Skin	125	87.4
Stage at diagnosis		
Local	61	40.6
Regional	24	16.8
Distant	58	40.6
Chemotherapy		
None	115	80.4
Chemotherapy, single agent	19	13.3
Chemotherapy, multiple agents	9	6.3
Radiation therapy		
No radiation	93	65.0
Radiation	50	35.0
HIV status		
HIV-positive	75	52.4
HIV-negative	68	47.6

**Table 2:** Median, two- and five-year survival rates for demographic and clinical variables in a population of Kaposi's sarcoma patients diagnosed at Mount Sinai Medical Center in Miami Beach, FL, USA.

Variable	Median survival (months) (95% CI)	*P* value*	2-year survival (%)	5-year survival (%)
Sex		0.84		
Female	28 (3, 106)		57.1	21.4
Male	23 (14, 26)		45.0	20.9
Race and ethnicity		0.01		
Non-Hispanic, white	20 (12, 25)		39.5	18.6
Hispanic white	56 (26, 128)		70.0	32.5
Black	8 (2, 23)		23.5	5.9
Smoking status		0.32		
Nonsmoker	23 (13, 31)		47.5	19.7
Ex-smoker	17 (8, 28)		43.2	21.6
Current smoker	36 (13, 134)		61.9	28.6
Unknown	15 (4, 26)		33.3	16.7
Age at diagnosis, years (range)		0.34		
21–40	22 (10, 26)		43.0	28.0
41–60	18 (9, 26)		39.0	22.0
61–80	38 (4, 128)		61.0	46.0
>80	32 (12, 88)		65.0	31.0
Year of Kaposi's diagnosis		0.004		
1987–1996	14 (9, 23)		35.0	12.0
1997–2007	87 (38, —)		65.0	56.0
Site of initial presentation		0.04		
Skin	23 (20, 28)		48.0	29.0
Nonskin	9 (3, 26)		30.0	29.0
Stage at Diagnosis		0.17		
Local	32 (14, 76)		54.1	27.9
Regional	22 (7, 123)		50.0	16.7
Distant	17 (9, 24)		36.2	15.5
Chemotherapy		0.26		
None	23 (18, 29)		47.8	23.5
Chemotherapy, single agent	14 (5, 52)		47.4	10.5
Chemotherapy, multiple agents	9 (3, 24)		22.2	11.1
Radiation therapy		0.98		
No radiation	23 (12, 31)		46.2	22.6
Radiation	22 (12, 28)		46.0	18.0
HIV status		0.91		
HIV-positive	19 (9, 26)		41.3	22.7
HIV-negative	25 (21, 32)		51.5	19.1

*Log-rank and Wilcoxon tests.

**Table 3:** Univariate and multivariate Cox-proportional hazards models in a population of Kaposi's sarcoma patients diagnosed at Mount Sinai Medical Center in Miami Beach, FL, USA.

Variable	Hazard ratio (95% confidence limits)	*P* value
Age		
>50	0.91 (0.60, 1.21)	0.66
<50*	1	
Year of diagnosis		
1997–2007	0.38 (0.24, 0.60) 1	<0.0001
1987–1996*	0.33 (0.19, 0.55)** 1	<0.0001
Sex		
Male	0.94 (0.69, 1.19)	0.84
Female*	1	
Ethnicity		
Hispanic, white	0.52 (0.36, 0.68) 1	0.01
Non-Hispanic, white*	0.55 (0.33, 0.90)** 1	0.02
Smoking		
Current	0.62 (0.46, 0.77)	0.17
Former	1.06 (0.73, 1.39)	0.82
Nonsmoker*	1	
Site of initial presentation		
Skin	0.69 (0.49, 0.88)	0.22
Nonskin*	1	
Stage		
Regional	1.14 (0.83, 1.45)	0.68
Distant	1.48 (0.99, 1.96)	0.07
Local*	1	
Chemotherapy	1.27 (0.92, 1.62)	0.43
No chemotherapy*	1	
Radiation	1.01 (0.67, 1.34)	0.98
No radiation*	1	
HIV status		
Positive	1.02 (0.67, 1.36)	0.91
Negative*	1	

*Reference level.

**Significant variables based on multivariate analysis.
